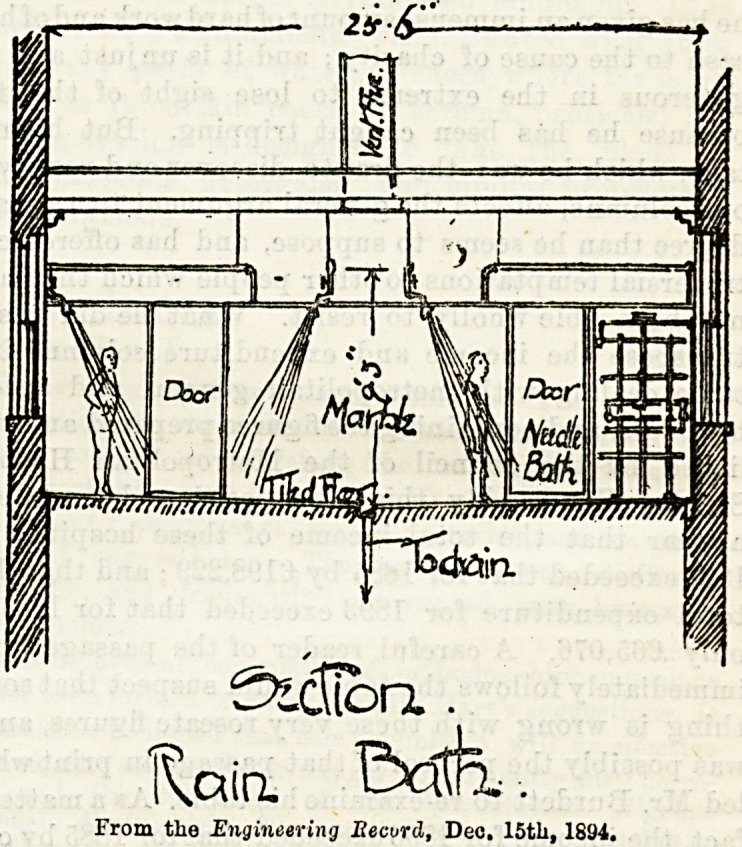# Rain Baths

**Published:** 1895-01-26

**Authors:** 


### PRACTICAL DEPARTMENTS.

### RAIN BATHS.

The *Engineering Record* last month contained a detailed account, with illustrations, of the new "Rain Baths" recently instituted at the Utica State Hospital for the Insane, under the superintendence of and from plans by the writer, William Paul Gerhard, C.E., consulting engineer for sanitary works. As such baths have not yet, we believe, been introduced into any English hospital or asylum, it may interest readers of The Hospital if we here give some idea of their plan and system and of their advantages, as set forth by Mr. Gerhard.

The idea of substituting douche or spray baths for the usual bath tubs in public institutions and bath-houses originated in Germany, and was first adopted in the United States not many years since. A rain bath was built first by the New York Juvenile Asylum, for the use of the children, at the suggestion of Dr. S. Baruch, a hydropathic physician, who published in 1891 a pamphlet, "A Plea for Public Baths." In due course other public bath-houses have adopted the plan, amongst which may be mentioned that erected by the trustees of the Baron de Hirsch Fund in the Centre Market Place, New York. Descriptions of some of these baths by Mr. Gerhard have been reprinted in pamphlet form, and this pamphlet was sent at the request of the New York State Commission in Lunacy to the medical superintendents of all the State hospitals in the State of New York. In reply to a question as to spray baths contained in a subsequent circular of inquiry from the New York State Commission in Lunacy addressed to asylum authorities, answers were received which were on the whole very favourable to the new system of bathing. The superintendent of the Willard State Hospital thus summarises its advantages over the ordinary bath.

"1. It is absolutely safe. There is no possibility of scalding a patient, and the more remote danger of suicide in a bath tub is also overcome.

"2. Cleanliness is assured and the temptation offered to lazy attendants to bathe more than one patient in the same water is removed.

"3. A great amount of time is saved, which under the old method is used in filling and emptying the tub. Our experience has been that one spray will do the work of two tubs in a little more than half the time.

"4. There is much less hot water used, and there is a consequent reduction in the amount of coal consumed. While we have made no accurate experiments in this respect, my opinion is that the saving is considerable."

In 1893 Mr. Gerhard was requested by the authorities of the Utica State Hospital to propose plans and estimate for fitting up a patients' bath-house in a building which had formerly been the bakery of the establishment, and in the following year this work was completed, and the experiment pronounced to be in every way a success.

The bath-house is a two storey structure, the ground floor being occupied by the bath-room proper, 30 ft. by 25½ ft., and a dressing-room 25½ ft. by 21 ft. Separate entrances, stairs, and vestibule are provided for male and female patients. There are also two private entrances. The staircases are of iron, 4 ft. wide. The building is situated in the rear of the large open court or quadrangle of the hospital, and can be reached from all the wards, under cover.

The bath-room contains four rows of sprays and douches, 30 overhead douches, and nine hand sprays. Down the centre of the floor runs a gutter for the removal of waste bath water, the floor being properly sloped on either side, as shown in the accompanying section, which we reproduce from the *Engineering Record* of December 1st. There are four lines of warm water supply pipes, three of which provide 10 inclined douches each, the fourth supplying a "needle bath,"

From the *Engineering Record*, Dec. 15th, 1894.

and the nine hand sprays. The room is lighted with eight large windows, fitted with opaque glass, while there are three in the dressing-room. Both rooms are fitted with incandescent electric lamps suspended from the ceiling. The bath-room walls are wainscotted with white Italian marble to a height of 6 feet, all sharp corners being rounded off to avoid possible bruising to the patients. A marble partition divides bath-room from dressing-room; the floors of both are laid with encaustic embossed "Alhambra" tiles.

The dressing-room is fitted with clothes boxes, a "dumb waiter" to carry up soiled garments to the "assorting room" on the second floor, which communicates with the laundry, and with water-closet and urinal. Benches are arranged round three sides and in the centre, and benches have also been placed in the bath-room itself, to enable the bathers to sit down while washing their feet. Cork mats are used in the dressing-room to keep the floor dry and the bather's feet warm. Both rooms are heated from overhead steampipes, to avoid any danger of scalding by contact with radiators or pipe coils, and pipes carried underneath in the excavated space near the bath-house serve to warm the tiled floor. Sanitary arrangements and ventilation appear to be carefully carried out.

It is estimated that, "allowing five minutes for undressing, fifteen minutes for bathing and drying, and ten minutes for dressing, each bath would occupy thirty minutes; hence, as there are 39 douches and sprays, 78 patients can be bathed per hour, or in five hours per day 390, approximating 400 patients. The amount of water used for each bather would be in ten minutes about 25 gallons, or 25 × 78, equaling 1,950 gallons per hour, or 9,750 gallons in five hours for bathing 390 patients."

The hot water apparatus is called a "gegenstrom" apparatus, and was manufactured by H. Schaffstaedt, of Giessen, Germany. The rest of the work has been carried out by American firms.

It will be interesting to note whether in future this system will find favour among English asylum authorities. The expense of such a departure in the first instance will no doubt prevent its adoption to any extent, but it remains to be seen how far its practical recommendations will in time overcome this initial difficulty.

## Figures and Tables

**Figure f1:**